# Preclinical outcomes of Intratumoral Modulation Therapy for glioblastoma

**DOI:** 10.1038/s41598-018-25639-7

**Published:** 2018-05-08

**Authors:** Andrea R. Di Sebastiano, Andrew Deweyert, Simon Benoit, Erin Iredale, Hu Xu, Cleusa De Oliveira, Eugene Wong, Susanne Schmid, Matthew O. Hebb

**Affiliations:** 1 Department of Clinical Neurological Sciences, Schulich School of Medicine and Dentistry, Western University, London, Ontario, Canada; 2 Department of Medical Biophysics, Schulich School of Medicine and Dentistry, Western University, London, Ontario, Canada; 3 Department of Anatomy and Cell Biology, Schulich School of Medicine and Dentistry, Western University, London, Ontario, Canada

## Abstract

Glioblastoma (GBM) is the leading cause of high fatality cancer arising within the adult brain. Electrotherapeutic approaches offer new promise for GBM treatment by exploiting innate vulnerabilities of cancer cells to low intensity electric fields. This report describes the preclinical outcomes of a novel electrotherapeutic strategy called Intratumoral Modulation Therapy (IMT) that uses an implanted stimulation system to deliver sustained, titratable, low intensity electric fields directly across GBM-affected brain regions. This pilot technology was applied to *in vitro* and animal models demonstrating significant and marked reduction in tumor cell viability and a cumulative impact of concurrent IMT and chemotherapy in GBM. No off target neurological effects were observed in treated subjects. Computational modeling predicted IMT field optimization as a means to further bolster treatment efficacy. This sentinel study provides new support for defining the potential of IMT strategies as part of a more effective multimodality treatment platform for GBM.

## Introduction

Glioblastoma (GBM) is the most common and aggressive primary brain cancer in adults, affecting ~1 in 33,333 people annually and ending the life of an untreated patient within 3 months after diagnosis^1^. The current standard of care is surgical resection, when possible, along with radiation and temozolomide (TMZ) chemotherapy. Despite maximum available treatment, the cancer inevitably recurs and yields a median survival of only 14 months^1^. There is now mounting evidence that electrotherapy may offer new promise as an effective treatment modality much needed for GBM patients^2^. The rationale for this strategy is based on an innate sensitivity of GBM cells to non-ablative electric current or fields that are innocuous to normal neural tissues, thus creating a putative safety margin for therapeutic development^3,4^. Such low intensity electrotherapy likely works through multiple mechanisms that relate, in part, to disruption of polarized cellular elements necessary for cytokinesis as well as changes in membrane permeability and channel homeostasis^5,6^. There is presently a single electrotherapy approved for GBM in the United States. This system includes a portable electric field generator to deliver low intensity (1–3 V/Cm), intermediate frequency (200 kHz) alternating electric fields, called tumor treating fields (TTFs), to supratentorial brain regions. TTFs are administered through arrays of insulated electrodes adhered to the patient’s scalp^7^. Continuous, long-term application is recommended and the transducer arrays are replaced every few days. The clinical impact of TTFs in GBM has been evaluated in two randomized, multi-centre trials^8,9^. The studies had methodological constraints but produced results supporting a significant clinical benefit in both recurrent and newly diagnosed disease. Potential drawbacks of TTF therapy include the need for a shaved head, frequent electrode changes, scalp complications from chronic electrode wear, stigmata of an external treatment device, inability to target infratentorial or spinal disease and high treatment-related costs^10,11^.

The most common (>90%) pattern of GBM progression occurs as aggressive, continuous extension from the site of the original lesion^12–15^. This important recognition has led to decades of research attempting to develop effective locoregional strategies to control growth of inoperable tumors and prevent recurrence following surgical resection^16–19^. Our group has been pioneering a novel locoregional electrotherapy, called Intratumoral Modulation Therapy (IMT), with the premise that an internalized electric field will exploit GBM electrosensitivity to greater advantage and with fewer limitations than an externally applied (e.g., scalp-mounted) source^3^. Still in the proof-of-concept stage, the clinical vision of IMT uses special purpose, magnetic resonance imaging (MRI)-compatible bioelectrodes strategically positioned within, or adjacent, tumor-affected regions of the central nervous system (CNS). A key feature of IMT is the ability to reach any aspect of the CNS to provide focused, titratable therapy directly within areas of disease. Bioelectrodes could be designed for personalized and comprehensive treatment coverage of GBM resection beds or non-operated lesions within eloquent or deep-seated CNS regions. The proximity of the IMT field source to GBM pathology will permit a broad, versatile spectrum of stimulation parameters custom optimized to tumor location and treatment response. Such a concealed, indwelling system is expected to support patient quality of life while providing sustained, low maintenance therapy that potently complements radiation and ongoing chemotherapeutic options.

We recently described an *in vitro* IMT approach using monophasic, low amplitude (4 V), low frequency (130 Hz) square wave pulses that induced apoptosis in patient GBM cells without notable impact on primary neurons^3^. Adjuvant IMT significantly increased GBM cell death when combined with TMZ or oncogene-targeting therapies^3^. These early results confirmed GBM sensitivity to directly applied, non-ablative electrical pulses but the low frequency parameters posed risk of neuronal entrainment and off-target neurological effects if applied within eloquent CNS areas^20^. The objective of the present study was to evaluate a novel profile of IMT parameters using intermediate frequency (200 kHz) stimulation with low risk of neuronal entrainment and a sinusoidal waveform to deliver continuous, alternating polarity, low intensity (±2 V) electric fields. These new parameters were first validated using our established *in vitro* methods then translated to test IMT for the first time in a customized *in vivo* GBM model.

## Results

### Intermediate frequency IMT selectively kills GBM cells and provides cumulative anti-neoplastic effects when administered with TMZ chemotherapy

**Figure 1:**
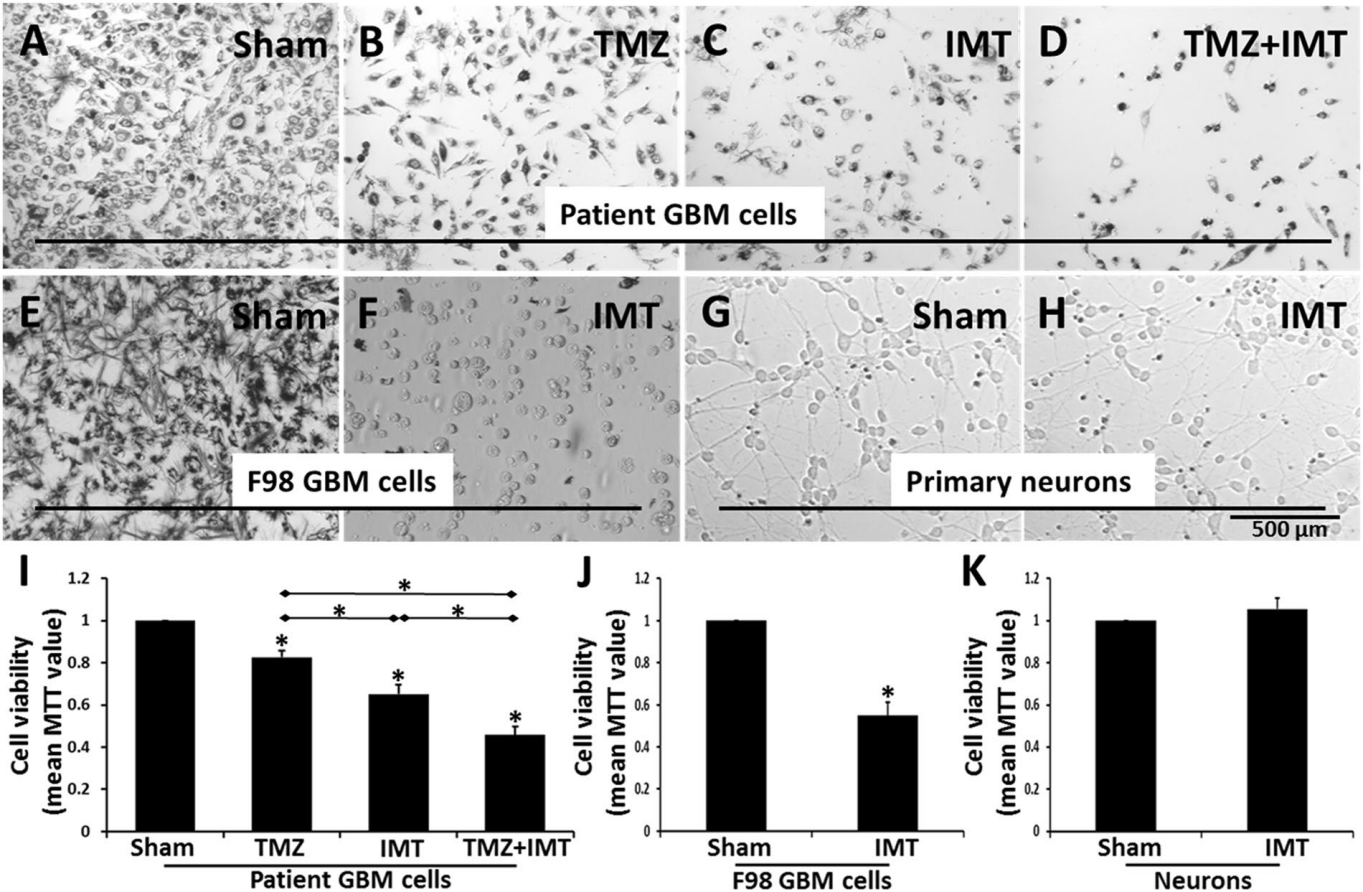
Impact of intermediate frequency IMT on GBM cells *in vitro*. Brightfield photomicrographs of patient and rodent F98 GBM cells and primary neurons are shown following a 3-day exposure to the indicated treatments. The images captured cell fields adjacent to the bioelectrode implanted within the cultures (not shown). (**A**) Patient GBM cells stained with the dark MTT chromogen demonstrate robust viability under sham conditions. A moderate reduction in staining, accompanied by pyknosis and decreased cell density was observed with (**B**) TMZ or (**C**) IMT monotherapy. (**D**) Concomitant TMZ+IMT resulted in a dramatic loss of cell viability compared to that achieved with either treatment alone. (**E**,**F**) Susceptibility to the same stimulation parameters was also evident in rodent F98 GBM cells as shown by MTT staining. (**G**,**H**) In contrast, the morphology and density of post-mitotic primary neuronal cultures, as shown here following trypan blue exposure, was not notably affected by IMT. (**I**) The viability of GBM cells from 3 different patient tumors was significantly reduced by TMZ or IMT alone. A cumulative GBM cell loss was produced by the combination of both treatments, likely reflective of distinct mechanisms of action. Asterisks directly above the data bars indicate a significant (P < 0.001) difference from the sham control. The significance (P < 0.001) between indicated data pairs is depicted by an asterisk above the horizontal lines. (**J**) F98 GBM (N = 3; P = 0.008) but not (**K**) primary post-mitotic neuronal (N = 4; P = 0.091) cultures were susceptible to IMT. Asterisks directly above the data bars indicate a significant difference from the sham control. All data are presented as mean ± standard deviation.

The MTT assay generated spectrophotometric values that reflected cell viability. Results of each treatment condition were normalized to those of the sham group. GBM cells from 3 patient tumors were independently treated and the data pooled for analysis. The individual MTT values are provided in Table 1. TMZ produced a modest but significant reduction in patient GBM cell viability to 82.6 ± 3.0% (P = 0.001), whereas IMT resulted in a greater drop to 65.1 ± 4.0% (P < 0.001) of sham values. Co-administration of TMZ with IMT resulted in a dramatic cumulative loss of GBM cell viability to 45.8 ± 4.0% (P < 0.001) of sham values (Fig. 1). The difference in GBM cell viability between TMZ and IMT (P < 0.001), and between TMZ (P < 0.001) or IMT (P < 0.001) and the combined TMZ + IMT treatments were all highly significant. The F98 GBM cells were similarly vulnerable to IMT, with a reduction to 54.9 ± 6.3% (N = 3; P = 0.008) of sham values. In contrast, primary neuronal cultures did not exhibit obvious changes in morphology nor were the MTT measures notably affected by IMT, with a mean group viability of 105.5 ± 5.3% (N = 4; P = 0.091) of sham values (Fig. 1).

**Table 1:** Spectrophotometric viability (MTT) analysis in patient GBM cells.

GBM #	Temozolomide	IMT	Temozolomide + IMT
1	0.79	0.68	0.49
2	0.85	0.67	0.47
3	0.84	0.59	0.41

Shown are the MTT values, normalized to the respective sham control, for GBM cells obtained from 3 patients. Cells were exposed to the indicated conditions for 3 days preceding analysis. IMT was statistically superior to temozolomide in reducing GBM cell viability and the combination of temozolomide and IMT produced dramatic, highly significant anti-neoplastic effects. These data are summarized in Fig. 1.

**Figure 2:**
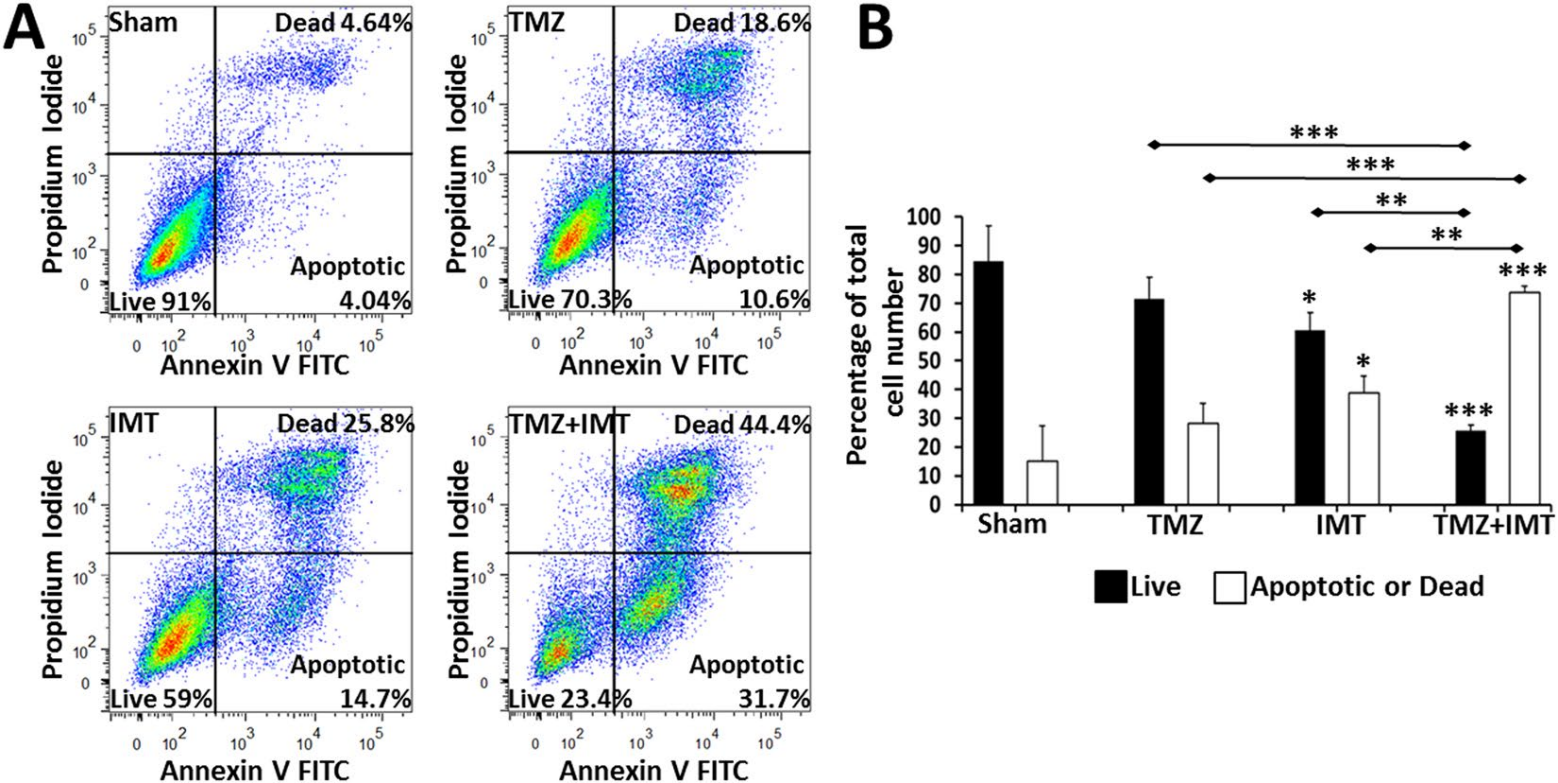
Flow cytometry measures of IMT response in patient GBM cells. (**A**) Representative scatterplots of annexin and propidium iodide (PI) labeling of apoptotic and dead patient GBM cells, respectively, after a 3-day exposure to the indicated treatment. IMT produced a greater cytotoxic effect than TMZ but the number of apoptotic and dead GBM cells rose dramatically with combined therapy. (**B**) Quantitative flow cytometry revealed a significant difference (P < 0.001) between live and apoptotic/dead fractions within each of the sham, TMZ and TMZ+IMT groups, but not in the IMT group (P = 0.053). This corresponded to a significant loss of GBM cell viability with IMT, but not TMZ, and a cumulative increase in cytotoxicity with the combination of the two treatments. These studies were performed in triplicate using primary GBM cells from 3 different patients. Asterisks directly above the data bars indicate a significant difference from the respective sham value (e.g., live compared to live). Significance between indicated data pairs is depicted by the asterisk(s) above the horizontal lines. Data are presented as mean ± standard deviation with significance indicated at P *< 0.05, **< 0.01 and ***< 0.001.

Flow cytometry with Annexin V and PI labeling permitted quantification of live, apoptotic and dead GBM cell fractions. Primary neurons were not able to withstand the flow cytometry methods. The assay was conducted on 15–30,000 GBM cells for each treatment condition using biological replicates from 3 patients. The values for apoptotic and dead cells were combined into a single category. The flow cytometry revealed 15.1 ± 12.2% apoptotic/dead GBM cells in the sham group compared with a progressive increase in this fraction following treatment with TMZ (28.2 ± 7.1%; P = 0.488), IMT (38.8 ± 6.0%; P = 0.032) and the combination TMZ + IMT (73.7 ± 2.1%; P < 0.001; Fig. 2; Table 2).

**Table 2:** Individual flow cytometry measures of IMT response in patient GBM cells.

GBM #	Sham		Temozolomide		IMT		Temozolomide + IMT	
	Live	Apoptotic or Dead	Live	Apoptotic or Dead	Live	Apoptotic or Dead	Live	Apoptotic or Dead
1	91.0	8.68	70.3	29.2	59	40.5	23.4	76.1
2	92.5	7.49	79.4	20.6	67.5	32.1	27.04	72.8
3	70.4	29.26	64.8	34.7	55.6	43.7	26.7	72.2

Shown are the percentages (%) of live (Annexin−/PI−) versus apoptotic (Annexin+/PI−) or dead (Annexin+/PI+) patient GBM cells quantified with flow cytometry. Each condition was evaluated in 3 biological replicates with 15,000–30,000 patient GBM cells exposed to the indicated treatment for 3 days. The differences in therapeutic impact between groups were highly significant and are summarized in Fig. 2.

### IMT significantly attenuates GBM growth ***in vivo***

**Figure 3:**
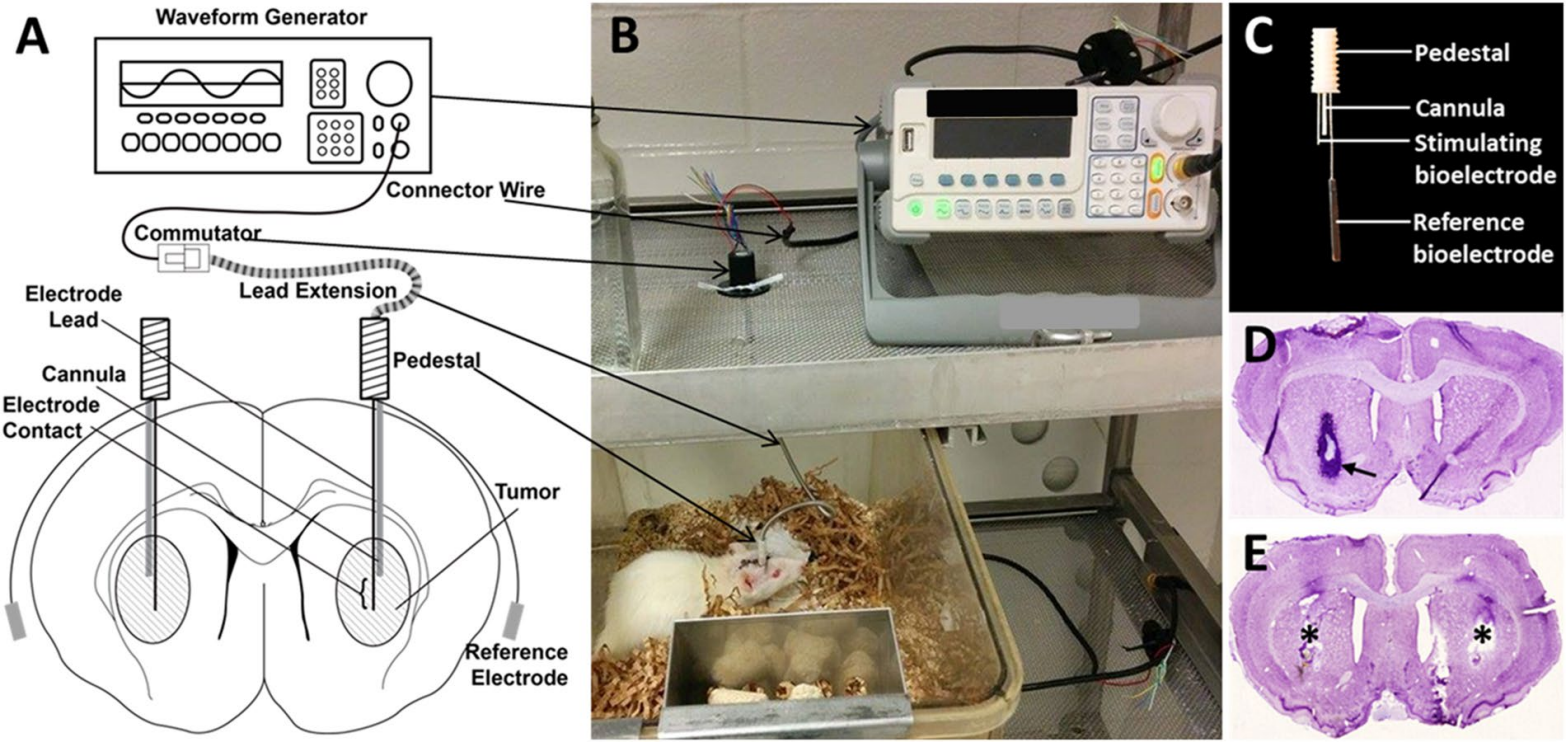
*In vivo* model to evaluate IMT in GBM. (**A**) Schematic and (**B**) photographic depiction of the *in vivo* IMT model. The animals could roam freely throughout the home cage. Bilateral GBM tumors were randomized so that one would receive sham treatment (i.e., hardware but no stimulation) and the other IMT between postoperative days 4–11. This provided an internal control tumor for every subject. (**C**) The IMT construct consisted of a lead with a single 1-mm stimulating bioelectrode, a cannula to deliver the GBM cells and a reference bioelectrode. The stimulating bioelectrode was located at the core of the GBM tumors and the reference bioelectrode positioned extracranially under the scalp. (**D**) Representative thionine-stained section of a control brain demonstrating a 4-day GBM, reflecting the tumor size when IMT was initiated (arrow). (**E**) Representative thionine-stained section through another control brain to show the effects of IMT on normal parenchyma. There was no tumor in this subject however bilateral IMT constructs were placed for delivery of 1 week of sham (left) or IMT (right). Symmetric hardware defects were evident (asterisks) without overt parenchymal injury from the stimulation.

The syngeneic rodent F98 GBM model was customized to evaluate IMT efficacy against GBM in the living brain (Fig. 3A–C). The treatment period spanned postoperative days 4–11 during which no neurological, behavioral or pain symptoms related to the stimulation were observed. Of the original 25-animal cohort, 15 animals were included in the final analysis. The excluded animals were: 4 with evidence of overt infection extending into the brain, 1 with insufficient tumor take, 3 with incomplete histological preparations and 2 that expired from undetermined causes. The first death occurred on postoperative day 3, prior to initiating IMT. The second death occurred 3 days after IMT initiation. The brain of this animal was examined histologically and no evidence of treatment-related complication (e.g., electrolysis, hematoma) was identified. In addition to the 25-animal cohort, control animals with unilateral tumor-only implants (N = 4) evaluated on postoperative day 4 (i.e., the time point of IMT initiation) revealed the expected GBM growth consistent across the group (Fig. 3D). Animals that received IMT and sham treatments without previous GBM implants (N = 3) revealed no obvious stimulation-related injury to normal brain parenchyma (Fig. 3E).

**Figure 4:**
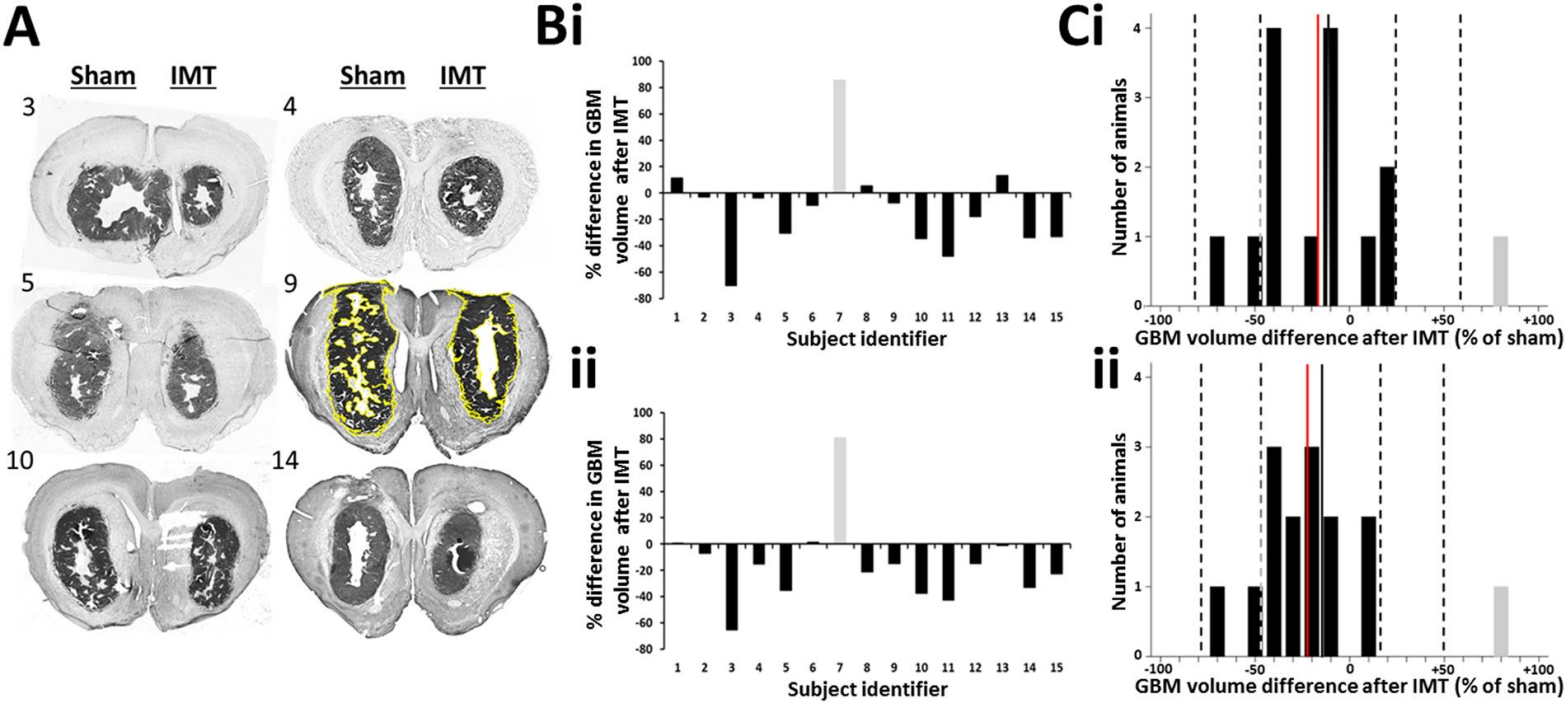
IMT attenuates locoregional GBM growth *in vivo*. (**A**) Representative, thionine-stained brain sections from 6 animals with GBM treated throughout this study. The 7-day course of IMT was randomized to the right or left GBM (treatment sides have been aligned in this figure for comparative purposes), with the contralateral tumor used as a sham control. The subject identifier is shown to the upper left of each section. GBM volumes were estimated by cumulative cross-sectional tumor areas quantified using ImageJ software, as demonstrated by the yellow tracing in subject 9. (**B**) The difference in estimated whole (i) and core (ii) GBM volume following IMT, relative to the internal sham control, is provided for every animal. Negative values are consistent with treatment response. The whole tumor measures incorporate serial sections through the rostrocaudal extent of the GBM whereas the core tumor measures were limited to the region surrounding the bioelectrode. There was one outlier (#7, shown in gray) with a reversal of expected effects, presumed technical in origin. (**C**) Distribution plots showing volume differences in the IMT-treated i) whole and ii) core tumor over 10% intervals. For example, a data bar on the −50% tick mark depicts the number of animals that had a 50–60% tumor reduction. The solid black-to-white lines indicate the cohort means when the data included the outlier shown in gray. The dashed lines indicate the standard deviation of this mean. Note that the outlier is well beyond 2 standard deviations of the mean. The red lines depict the cohort means with the outlier excluded.

**Figure 5:**
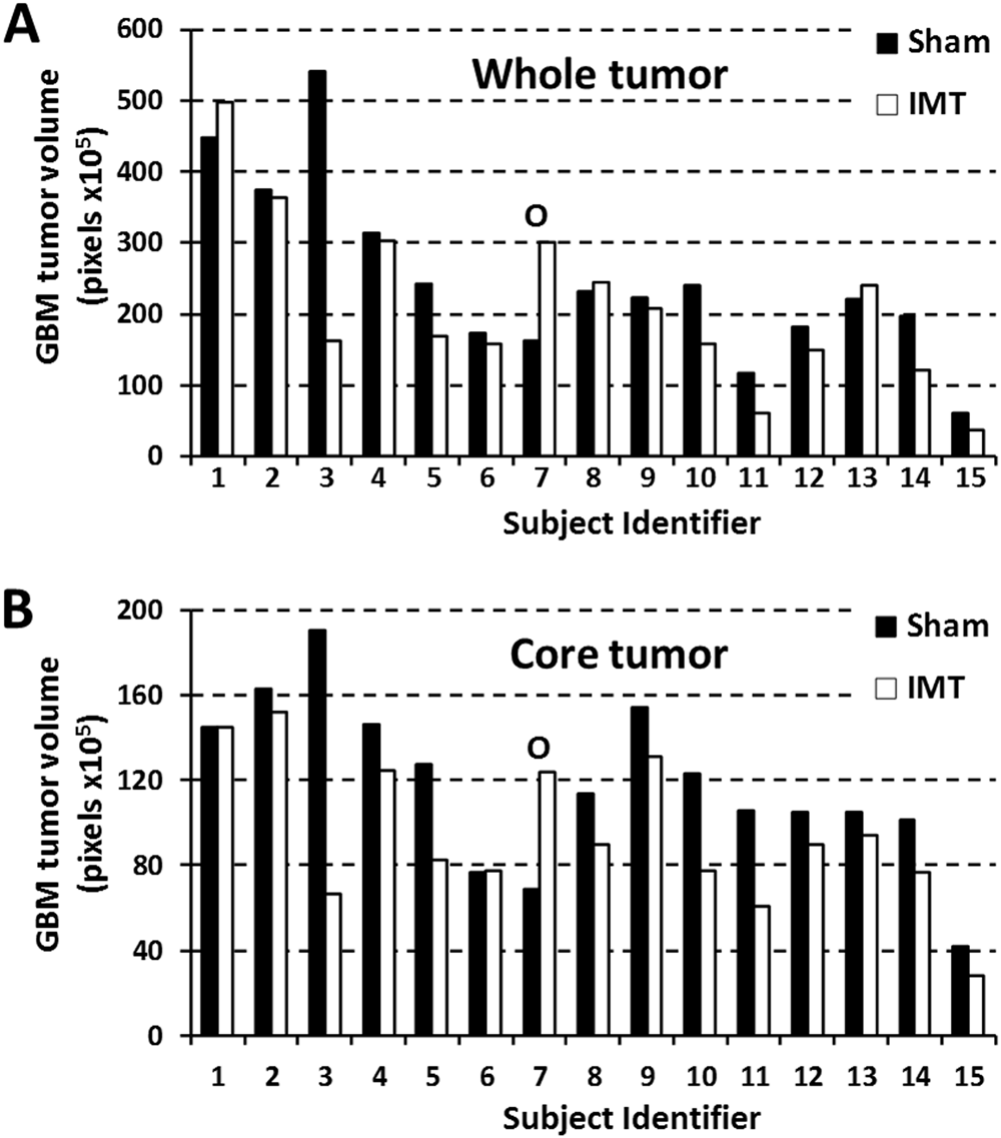
Paired *in vivo* GBM responses to sham and IMT conditions. Raw tumor measures calculated from the (**A**) whole tumor and (**B**) core tumor are shown after sham treatment (black) or IMT (white). The statistical outlier shown in Fig. 4 has been left in this dataset for completion (subject #7; labeled O). The IMT-treated GBM was smaller than the sham-treated tumor in 11 of 15 (73%) and 12 of 15 (80%) subjects using whole and core tumor measures, respectively.

The bilateral GBM tumors were randomized to sham treatment or IMT, with 8 right- and 7 left-sided tumors receiving IMT by the end of the study. GBM tumors that received IMT were typically more rounded and compact compared to the oblong, irregular configuration of sham-treated lesions. GBM volumes were estimated through the rostrocaudal extent of the tumor (‘whole tumor’) and also within a core region at the presumed centre of the IMT electric field (‘core tumor’). The whole tumor measures revealed smaller IMT-treated, compared to sham-treated, tumors in 11/15 (73%) animals. There was a single, statistically-proven outlier (subject #7) in which the discrepancy between IMT- and sham–treated tumors was drastically reversed. The reason for this finding was not identified, however hypothesized to be technical in origin, such as a problem with the GBM implant or the laterality marker. The change in tumor volume for this animal fell well past 2 standard deviations from the mean (i.e., >95th %-ile of the normal data distribution) and was identified by both the Dixon’s (P = 0.028) and Grubb’s (P = 0.016) tests as a significant outlier. The statistical evidence supported its exclusion; however the cumulative GBM measures for the entire cohort were calculated both in the presence and absence of the outlier subject (Figs. 4 and 5). With the outlier included, there was a non-significant 14.5% reduction in IMT-treated whole tumor volume (P = 0.107) that rose to a significant 19.5% reduction (P = 0.047) with the outlier excluded (Table 3). The percent change in the IMT tumor volume relative to that of the sham tumor was also calculated in each subject and averaged over the cohort. These data aligned with the cumulative tumor measure analysis and showed that with inclusion of the outlier, IMT produced a whole tumor reduction of 12.7 +/− 35.8% (P = 0.096). With subject #7 excluded, the volume reduction of the IMT-treated GBM increased to a highly significant 19.7 +/− 24.3% (P = 0.005).

**Table 3:** Cumulative *in vivo* tumor burden in sham and IMT-treated GBM within the 15-animal cohort.

	Cumulative whole tumor volume (pixels × 10^6^)		Whole tumor reduction with IMT (%)	P-value	Cumulative core tumor volume (pixels × 10^6^)		Core tumor reduction with IMT (%)	P-value
	Sham	IMT			Sham	IMT		
Outlier included	366.3	313.4	14.5	0.107	176.6	141.9	19.7	0.015
Outlier excluded	356.2	286.9	19.5	0.047	169.7	129.5	23.7	0.002

Core tumor analysis focused on the GBM region nearest the bioelectrode and identified 12/15 (80%) animals with smaller IMT-treated, compared to sham-treated, core tumors. Subject #7 was again identified as a statistical outlier using the Dixon’s (P = 0.004) and Grubb’s (P = 0.002) tests. Cumulative core GBM burden for the whole group was 19.7–23.7% smaller in IMT tumors compared to sham controls, a highly significant response regardless of the outlier measure (Table 3). There was also a significant mean individual reduction of 15.8 +/− 32.0% (P = 0.038) when assessed with the outlier included. This measure rose to 22.7 +/− 18.3% (P = 0.0002) with the outlier excluded.

### Computer simulation predicts IMT electric field properties in GBM tumor and brain

**Figure 6:**
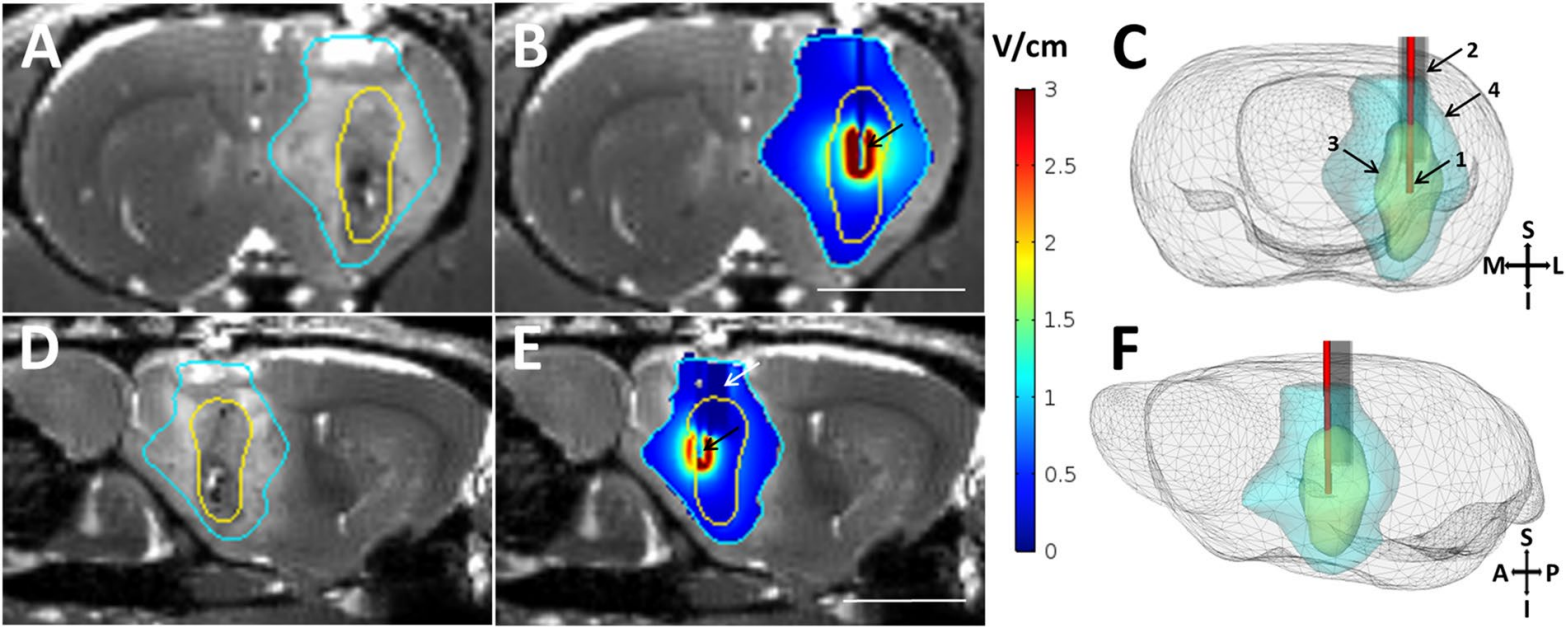
Image-based simulation of IMT electric fields. A 9.4T MRI system was used to generate true fast imaging with steady state precession (TrueFISP) images of a 7-day GBM tumor in the rodent brain. Coronal (**A**,**B**) and sagittal (**D**,**E**) images are shown. (**A**,**D**) Brainsuite software (v.16a1) was used to segment the tumor (yellow trace) and edema (turquoise trace). The segmented volumes were imported to COMSOL Multiphysics (v.5.2a), where the intratumoral bioelectrode and cannula geometries were simulated. (**B**,**E**) The conductance and dielectric values of the hardware (bioelectrode, black arrow; cannula, white arrow) and neural elements were used with the boundary condition of constant voltage amplitude of 2.0 V at 200 kHz for the bioelectrode and the reference electrode applied outside the skull. The resulting map of the electric field magnitude was exported and combined with the TrueFISP MR images for visualization in 3D Slicer (v.4.4.0). The electric field data within the tumor was analyzed using MATLAB R2015b to estimate the fraction of tumor receiving specific field amplitudes (see Fig. 7). Three-dimensional rendering of the rodent brain shown in (**C**) coronal and (**F**) oblique sagittal views depict the relationship between the 1) intratumoral bioelectrode, 2) cannula, 3) GBM mass and 4) region of tumor-associated cerebral edema. S, superior; I, inferior; M, medial; L, lateral; A, anterior; P, posterior. The scale bar in B corresponds to panels A and B; that in E to panels D and E; both depict 5 mm.

**Figure 7:**
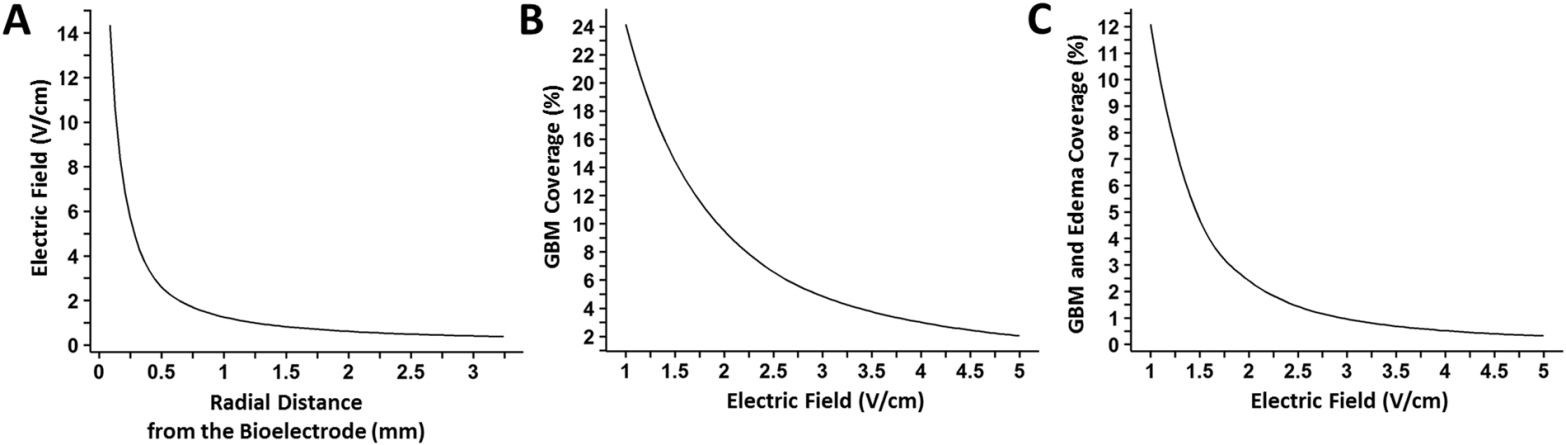
Predicted relations between IMT electric field amplitude, radial field dimensions and extent of GBM region coverage in the present *in vivo* treatment model.

These studies sought to define the extent of *in vivo* treatment coverage produced within the GBM environment using the present IMT hardware and stimulation parameters. IMT electric field modeling was performed in a representative 7-day rodent GBM to best estimate the tumor size and brain conditions at the mid-way point of the current IMT protocol which extended between days 4–11 following GBM cell implantation. The simulation analysis demonstrated a tightly constrained electric field centered near the core of the GBM mass with radial extension to a modest fraction of the tumor and surrounding edematous brain (Fig. 6). The amplitude of the IMT electric field decreased logarithmically with the radial distance from the bioelectrode. Field strengths of 4 V/cm, 3 V/cm, 2 V/cm and 1 V/cm extended to 0.32 mm, 0.43 mm, 0.63 mm, 1.24 mm, respectively, from the source. It was estimated that the fractions of GBM tumor that received an IMT electric field amplitude above these thresholds were 3.0%, 4.8%, 9.5% and 24.2% respectively. When combining the regions of GBM tumor and associated edematous brain, the fractions predicted to receive these field thresholds were 0.5%, 1.0%, 2.4% and 12.1%, respectively (Fig. 7).

## Discussion

This preclinical study is, to our knowledge, the first to demonstrate the efficacy of an implantable electrotherapy using non-ablative, intermediate frequency stimulation in patient-derived GBM cells and in a cohort of living animals with intracerebral GBM. Primary GBM cells, but not neurons, were exquisitely sensitive to low amplitude, sinusoidal pulses at a frequency out of range for neuronal entrainment or thermal injury^20,21^. IMT-mediated cytotoxicity was largely apoptotic and enhanced GBM cell death when co-administered with TMZ chemotherapy. This *in vitro* proof-of-concept was translated to an animal model in which a 1-week course of continuous IMT monotherapy produced a significant reduction of mean GBM volume in the living brain. These highly promising data likely do not reflect the full potential of IMT. There are technical and biological aspects of the present *in vivo* model which may have led to an underestimation of therapeutic efficacy. For example, the rudimentary, single-contact bioelectrode and applied stimulation parameters were predicted to generate an IMT electric field ≥1 V/cm (i.e., the lower threshold recommended for the external TTF device) throughout only ~24% of the estimated volume of a 7-day tumor^11^. The compact IMT field dimensions (i.e., ~1.24 mm radius for a ≥1 V/cm field) would poorly accommodate a suboptimally positioned bioelectrode, particularly if located near or beyond the GBM boundary, which may explain the inter-animal variability in treatment response. The measured therapeutic impact, while significant, was likely less than what may be achieved using hardware and stimulation parameters that provide comprehensive coverage of the tumor-affected region. The large GBM burden in the F98 model also created a greater treatment challenge than would be expected with small volume or recurrent/residual tumors. In our hands, a deposit of 40,000 F98 cells produced robust growth, expanding into moderate-sized lesions as early as day 4 and massive GBM tumors with cerebral edema and central necrosis by day 11 post-implantation. Others have reported a mean survival between 18–25 days in rats harboring these GBM tumors^22,23^. Anecdotally, the rate of growth may be proportionally greater than typical for GBM in humans, creating a highly formidable model in which to evaluate a putative new therapeutic device.

The development of an indwelling electrotherapy for GBM is predicated on a global experience using deep brain stimulation (DBS) devices engineered to treat non-oncological conditions, such as movement, pain, psychiatric and seizure disorders^24,25^. DBS entails stereotactic placement of in-line, multi-contact leads into target brain regions, controlled by a remote-accessed, implantable pulse generator. This technology typically delivers continuous, monophasic, square wave pulses at low amplitude (e.g., 1–5 V) and frequency (e.g., 90–185 Hz) to disrupt and entrain pathological firing patterns in a reversible, non-injurious manner^26^. DBS and IMT are starkly different in indication, operational parameters and hardware design. The electrical output of prospective IMT systems will be defined by non-ablative pulses coupled to customized and distinct profiles of waveform, polarity, and frequency parameters titrated to individual tumor response and location. This study utilized a low amplitude (+/−2 V), intermediate frequency (200 kHz), sinusoidal waveform with reversing polarity intended to maximally disrupt electrical homeostasis in GBM cells. The pulse frequency was 1000-fold higher than typical for DBS and well beyond the range of neuronal entrainment so not to incite adverse neurological effects when stimulating tumor-infiltrated CNS regions. This amplitude and frequency are also below the threshold for producing thermal injury and have been shown to selectively target GBM cells with relative safety in preclinical studies and human TTF therapy^3,4,7,21^. The irregular and variable anatomy of GBM disease also mandates unique IMT hardware design. Rather than a single, in-line row of contacts typical of DBS systems, comprehensive tumor coverage requires bioelectrodes with versatile, multi-dimensional configurations driven by a field generator capable of producing a unique spectrum of IMT parameters. DBS has traditionally used a voltage-driven treatment platform, however newer devices are available that utilize constant current parameters with or without directional control^27,28^. Tight regulation of current flow within disease-affected brain regions may provide greater therapeutic impact when the goal is to re-program pathological firing patterns in non-oncological disease; however, it remains unknown whether the same logic applies to electrotherapies designed for CNS cancer. Conductive (uninsulated) bioelectrodes may be powered by a voltage-driven system with the resultant current flow gated by local tissue resistivity. The heterogeneous architecture of the brain and GBM tumors would result in constant voltage with variable and dynamic current flow. In contrast, the same bioelectrodes powered by a constant current platform could provide a defined rate of flow maintained by feedback modulation that adjusts the voltage to accommodate varying resistivity. A third option for IMT delivery is to establish an intratumoral electric field without current flowing between the bioelectrodes and the tissues using non-conductive (insulated) bioelectrodes within the GBM-affected region. In the present work, IMT was voltage-driven with a peak-to-peak pulse amplitude of 4 volts (±2 V) which did not elicit overt behavioral changes, seizures or focal neurological deficits, nor histological evidence of electrolysis in the brain or tumor. As a pilot investigation, a small fraction (2/15) of the cohort received insulated bioelectrodes with other IMT parameters remaining constant. There was a positive treatment response in these animals measured by both whole and core tumor analysis, indicating that an applied intratumoral electric field may be sufficient to attenuate tumor growth. Further studies are needed to define the properties of conductive versus non-conductive implanted bioelectrodes in GBM disease.

This study demonstrated the in vitro efficacy of intermediate frequency IMT in patient GBM cells and showed that a rudimentary, pilot IMT device could significantly reduce GBM volume across a 15-animal cohort. This is the first demonstration that GBM tumors may be therapeutically impacted using an implantable system to chronically deliver low intensity, non-ablative electric fields. The current data provide strong justification to develop IMT hardware and application settings that provide more comprehensive tumor coverage and, hopefully, enhanced therapeutic benefit. Successful advances in IMT technology could lead to a novel treatment modality desperately needed for GBM and other high fatality CNS cancers.

## Materials and Methods

### GBM cell cultures

These protocols were approved by the Human Research Ethics Board and the Animal Care Committee at Western University and carried out in accordance with the Tri-Council Policy for research involving human subjects and the Canadian Council on Animal Care. Informed consent was obtained from all patients or their legal guardians prior to using their GBM tissue in this study. Human GBM cells were isolated from operative tumor specimens obtained from 3 patients (39 y male, 44 y female, 65 y female). Specimens were not genetically screened for study purposes. The tumor samples were collected into phosphate-buffered saline (PBS) with 0.5% fetal bovine serum (FBS; Life Technologies) at the time of surgery. The tissue was digested and filtered then suspended in Dulbecco’s modified Eagle’s medium (DMEM; Wisent Bioproducts) supplemented with 10% FBS, 1% non-essential amino acids and 1% penicillin/streptomycin (Life Technologies). Homogenates were plated on a 35 mm dish for 30 minutes to allow for separation of blood cells. The upper cell suspension was then transferred to two wells of a 24-well plate, freshly pre-coated with 10 µg/ml poly-L-lysine (Trevigen Inc) and incubated at 37 °C with 5% CO_2_. Cultures were passaged at approximately 80% confluence and split 1:2 using 0.25% trypsin with 0.53 mM ethylenediaminetetraacetic acid (EDTA; Wisent). The medium was changed twice per week. All assays were conducted using GBM cells from cultures at passages 4 through 12.

The F98 GBM cell line (CRL2397™; ATCC) was originally created following a single N-ethyl-N-nitrosourea injection to a pregnant Fischer rat to produce offspring with malignant glioma tumors that were then maintained in culture^22,23^. The low immunogenicity of F98 cells in syngeneic Fischer rats offers advantages over xenogeneic and allogeneic models that require immunosuppression or may exhibit spontaneous tumor regression. F98 GBM tumors are highly proliferative, invasive and recalcitrant to conventional therapies, closely mimicking the human disease. F98 cells were cultured in 100 mm dishes in DMEM supplemented with 10% FBS, 1% non-essential amino acids and 1% penicillin/streptomycin (Life Technologies) in a humidified incubator at 37 °C with 5% CO_2_. The medium was changed twice per week and cultures passaged at ~80% confluence and split 1:2 using 0.25% trypsin with 0.53 mM EDTA (Wisent).

### Primary neuronal cultures

IMT was evaluated in post-mitotic primary neurons isolated from embryonic rat brains (N = 3). Cerebral cortices from E18 Wistar embryo were dissected into a 14 ml conical tube containing 1.8 ml of Hank’s balanced salt solution (HBSS; Wisent) and centrifuged at 4000 × g for 1 min at room temperature. HBSS was aspirated and 1.8 ml of solution A containing 5 ml HBSS, 6 μl MgSO_4_ (1 M) and 2 ml trypsin (Sigma Aldrich) were added. The tube was mixed well and placed in an automated rotator at 37 °C for 25 minutes. After rotation, 3.6 ml of solution B containing 7 ml HBSS, 8 μl MgSO_4_ (1 M), 175 μl DNase1 (10 mg/ml) and 112 μl trypsin inhibitor (100 μg/ml; Roche Life Sciences) were added and mixed for 2 minutes, centrifuged at 4000 × g for 5 min at room temperature, and then the HBSS was aspirated. Finally, 6 ml of solution C containing 20 ml of HBSS, 48 μl MgSO_4_ (1 M), 1.3 ml DNase1 (10 mg/ml), and 1 ml trypsin inhibitor (100 μg/ml) were added to the resulting cell pellet (Roche). These cells were transferred to a 50 ml falcon tube and another 6 ml of solution C was added. The cells were titrated, centrifuged at 4000 × g for 5 minutes and the supernatant aspirated. The cell pellet was re-suspended in 36 ml of media containing 96% neural basal media (Wisent), 2% B27 supplement, 0.8% N2 Supplement, 0.5% penicillin/streptomycin, 0.25% Glutamax (Life Technologies), and 0.1% Amphotericin B solution (Sigma Aldrich). Cells were counted with a hemocytometer, plated in 35 mm wells coated with 7% poly-L-Ornithine (Sigma Aldrich) at density of 0.5 × 10^6^ cells/well and kept in a humidified incubator at 37 °C with 5% CO_2_. On the third day of culture, the media was changed and wells were fitted with the IMT apparatus for delivery of 72 h of sham or IMT conditions.

### *In vitro* IMT model

The impact of IMT alone and combined with TMZ was independently evaluated in triplicate using primary human GBM cells, rodent F98 GBM cells and post-mitotic rodent primary neurons. GBM cells and primary neurons (1–5 × 10^5^ cells) were cultured in 35 mm wells in standard 6-well plates. GBM cells were grown to ~70% confluence before treatment. The IMT model was created by fitting each well with a clinical grade, platinum-based strip bioelectrode (AD-Tech) around the periphery and a single contact platinum-iridium bioelectrode (Medtronic Ltd.) at the center of the well and cell culture^3^. A waveform generator (Rigol DG1022; Electro-Meters Ltd.) was used to deliver biphasic sinusoidal pulses with low amplitude (+ /−2 V; peak-to-peak 4 V) and intermediate frequency (200 kHz) for a period of 72 hours. Control wells (i.e. sham-treated) were fitted with electrodes but no current was delivered. GBM cells treated with chemotherapy were plated with DMEM containing TMZ (50 μM; Sigma Aldrich) and received 72 hours of concomitant IMT or sham conditions. This TMZ concentration reflects clinically relevant levels corresponding to the *in vivo* plasma concentration of 150 mg/m^2^ in the adjuvant phase of GBM treatment^29^.

### Cell viability assays

Cell viability was evaluated using the 3-(4,5-dimethylthiazol-2-yl)-2,5-diphenyltetrazolium (MTT) spectral analysis (Sigma Aldrich). This spectrophotometric assay measures the reduction of yellow MTT by mitochondrial succinate dehydrogenase to an insoluble, dark purple Formosan product. Immediately following the GBM cell treatments described above, MTT (80 µl at 5 mg/ml) was added to the 35 mm wells and incubated for 3 hours at 37 °C in a humidified 5% CO_2_ atmosphere. The cells were then lysed to release the purple Formosan product by the addition of 600 µl dimethyl sulfoxide for 15 minutes at room temperature. Absorbance was measured using an enzyme-linked immunosorbent assay plate reader (Fisher Scientific). Cell viability was estimated using optical density values at 570 nm with references at 655 nm detected in each well.

Trypan blue exclusion was used as a confirmatory, qualitative measure of cell viability. Briefly, 0.1 ml of a 0.4% trypan blue solution (Lonza) was added for every 1 ml culture media and the cells incubated for 2 minutes at room temperature. Brightfield images of cells stained with MTT and trypan blue were obtained using a Motic AE31 inverted microscope fitted with an Infinity 1–3 scientific complementary metal-oxide semiconductor camera (Lumenera Corp).

An Annexin V Apoptosis Detection Kit with propidium iodide (PI; BioLegend) was used to quantify live, apoptotic and dead GBM cell fractions, as per the manufacturer’s instructions. Cell fractions were analyzed using a Becton Dickinson LSR II SORP flow cytometer running FACSDiva software (BD Biosciences). Cells were first gated on forward scatter (FSC−) versus side scatter (SSC−) characteristics before excluding doublets using consecutive gating FSC-Area versus FSC-Width and SSC-Area versus SSC-Width plots. The populations of annexin V+/PI−, annexin V+/PI+, annexin V−/PI+ and annexin V−/PI− were then calculated with quadrant gates. Approximately 15,000–30,000 single GBM cells were acquired per each of three patient samples at a maximum event rate of 5,000 events per second. Data were analyzed using FlowJo v 9.6.3 (TreeStar, Inc).

### *In vivo* GBM model and IMT

Bilateral orthotopic GBM tumors were established by implanting F98 cells into the brain of syngeneic adult female Fischer rats (Charles River)^22,23^. A cohort size of 25 was chosen to adequately temper inter-animal variability and account for unexpected problems or deaths. Subjects were maintained under isoflurane anesthesia for stereotactic implantation of chronic cannula-bioelectrode constructs (Plastics One) into bilateral striata (coordinates from bregma: anteroposterior 1 mm, lateral +/−3 mm, dorsoventral −6 mm). The constructs consisted of a 5 mm long steel cannula with 1 mm luminal diameter, running parallel to a 6-mm-long, rigid lead with a 1 mm long, 0.25 mm diameter bioelectrode contact at the distal tip. The reference bioelectrode was connected by a flexible 1 cm long insulated wire and positioned extracranially in the temporal or nuchal soft tissues. These three components were secured at a common, accessible pedestal cemented to the skull. A key advantage of this cannula-bioelectrode construct was the ability to implant GBM cells that form a large tumor mass centered around the IMT bioelectrode within the brain. In two animals (#10, 11), the bioelectrode contact was insulated with a thin layer of Entellan^®^ (Sigma Aldrich) prior to implantation. This pilot modification was intended to assess the anti-GBM effect of an intratumoral electric field without current flowing between the bioelectrodes and tissue.

F98 GBM cells (4 × 10^4^ in 2 µl DMEM) were delivered bilaterally through the implanted cannulae, after which the pedestal was covered with a protective cap. Two operators were present for quality assurance in all surgeries. On postoperative day 4, IMT was randomized to the right or left tumor and the respective bioelectrode was connected to a waveform generator (Rigol DG1022; Electro-Meters Ltd) via an extension cable and a commutator that permitted the animal to move freely throughout the home cage. The contralateral tumor served as an internal sham control (i.e., identical tumor and hardware implants but without stimulation). Continuous IMT monotherapy was delivered between postoperative days 4–11 using the *in vitro* parameters (i.e., sinusoidal waveform, +/−2V, 200 kHz). Three control subjects received bilateral hardware without GBM cell delivery to assess potential adverse effects of IMT on normal brain structure. Five subjects had unilateral implantation of GBM cells, as described above, using a Hamilton syringe and without IMT hardware. Four of these subjects were used to assess GBM size after 4 days of growth (i.e., the size at the onset of IMT). One animal underwent MRI on postoperative day 7 as part of the simulation studies, described below. All animals were given free access to food and water, perioperative antibiotics and analgesics, and monitored daily for medical or neurological complications. On postoperative day 11, animals were deeply anesthetized with sodium pentobarbital and transcardially perfused with 4% paraformaldehyde. The brains were extracted, cryoprotected and frozen. Brains were cut into 16–35 µm thick sections through the rostrocaudal extent of the tumors, mounted onto microscope slides and stained with thionine. Processed sections were digitally imaged with a Nikon Eclipse Ni-E microscope.

### GBM tumor analysis

Digitized brain images were imported into ImageJ v.2.0.0 software for tumor measurement by a blinded observer. The wand tracing tool was used to select the GBM tissue from surrounding brain with identical tolerance detection settings for the IMT- and sham-treated tumors. Tolerance detection ranged from 10–40 pixels in 8-connected mode and was adjusted accordingly between serial sections for variations in staining intensity. Cross-sectional areas in serial sections were quantified in pixels and summed to provide relative estimates of tumor volume. The whole tumor was measured using serial sections through the rostrocaudal extent of the GBM. A more focused measure of the tumor ‘core’ was calculated to assess the impact of IMT in the region of highest GBM burden (i.e., at the implantation site) and anticipated center of the IMT electric field. This core region was objectively defined within 4 brain sections containing the largest cross-sectional area of both sham- and IMT-treated tumors in addition to the two immediately flanking sections.

### IMT electric field simulation

The IMT electric field created by the treatment parameters used in this study was simulated based on 9.4 T MRI data from a representative control animal with a unilateral 7-day GBM, as described above. Imaging at this time point depicts the tumor size midway through the treatment period (i.e., day 4–11). MRI data was imported to Brainsuite v.16a1 for segmentation of the GBM tumor mass, associate cerebral edema, whole brain and skull. Segmented volumes were then imported to Multiphysics v.5.2a (COMSOL Inc.) where the intratumoral bioelectrode, reference bioelectrode and cannula geometry was simulated according to the *in vivo* IMT paradigm. The bioelectrode was modeled with stainless steel insulated with a 0.025 mm thick layer of polyimide except for the distal tip where a 1 mm contact was exposed. The conductance and dielectric properties of the segmented brain tissues, skull and bioelectrode apparatus were then specified within the COMSOL Electric Currents user interface which was employed to compute the electric field^30,31^. A constant voltage of 2.0 V at 200 kHz was applied across the intratumoral and reference bioelectrodes within this computer model. The resulting map of the electric field amplitude was exported and merged with the MR images for visualization in 3D Slicer v.4.4.0. The dimensions and amplitude of the electric field within the tumor and brain were analyzed using MATLAB R2015b (MathWorks Inc).

### Magnetic resonance imaging

MRI studies were performed at The Centre for Functional and Metabolic Mapping, Robarts Research Institute, Western University. An Agilent small animal 9.4 T MRI unit was used to obtain true fast imaging with steady state precession (TrueFISP; TR = 7 ms, TE = 3.5 ms, flip angle = 30 degrees, number of frequencies = 4, number of averages = 4, total acquisition time = 42 minutes, field of view = 28 mm × 28 mm × 28 mm, acquisition matrix = 140 × 140 × 140).

### Statistical analysis

A t-test or Wilcoxon signed-rank test was used to compare single and paired *in vitro* data sets that followed or deviated from a normal distribution, respectively. Multiple pairwise comparisons were performed using one-way analysis of variance (ANOVA) followed by Tukey post-hoc analysis. *In vivo* tumor measures were evaluated using the Dixon’s and Grubb’s tests for outliers. The *in vivo* IMT response was assessed by calculating the percent change in tumor volume for each subject and using a single sample t-test to compare the cohort values to a hypothetical mean change of zero percent. A complementary analysis did not assume uniform untreated rates of growth amongst tumors and compared the cumulative GBM burden following sham and IMT treatment within the entire cohort using a paired sample t-test. One-tailed analyses were used for statistical comparisons based on the known *in vitro* efficacy of IMT against GBM^3^. Statistical software used included SigmaStat (Systat Software Inc) and SPSS v 14.0 (IBM). Data are presented as mean ± standard deviation with significance assumed at p < 0.05.

### Data availability

All data analysed during this study are included in this published article.
